# sEMG-assisted inverse modelling of 3D lip movement: a feasibility study towards person-specific modelling

**DOI:** 10.1038/s41598-017-17790-4

**Published:** 2017-12-18

**Authors:** Merijn Eskes, Alfons J. M. Balm, Maarten J. A. van Alphen, Ludi E. Smeele, Ian Stavness, Ferdinand van der Heijden

**Affiliations:** 1 Dept of Head and Neck Oncology and Surgery, Netherlands Cancer Institute, Plesmanlaan 121, 1066 CX Amsterdam, The Netherlands; 2 MIRA Institute of Biomedical Engineering and Technical Medicine, University of Twente, Drienerlolaan 5, 7522 NB Enschede, The Netherlands; 3 Dept of Oral and Maxillofacial Surgery, Academic Medical Center, Meibergdreef 9, 1105 AZ Amsterdam, The Netherlands; 4 ACTA Academic Centre for Dentistry Amsterdam, Gustav Mahlerlaan 3004, 1081 LA Amsterdam, The Netherlands; 5 Dept of Computer Science, University of Saskatchewan, 176 Thorvaldson Building, 110 Science Place, Saskatoon, SK S7N 5C9 Canada

## Abstract

We propose a surface-electromyographic (sEMG) assisted inverse-modelling (IM) approach for a biomechanical model of the face to obtain realistic person-specific muscle activations (MA) by tracking movements as well as innervation trajectories. We obtained sEMG data of facial muscles and 3D positions of lip markers in six volunteers and, using a generic finite element (FE) face model in ArtiSynth, performed inverse static optimisation with and without sEMG tracking on both simulation data and experimental data. IM with simulated data and experimental data without sEMG data showed good correlations of tracked positions (0.93 and 0.67) and poor correlations of MA (0.27 and 0.20). When utilising the sEMG-assisted IM approach, MA correlations increased drastically (0.83 and 0.59) without sacrificing performance in position correlations (0.92 and 0.70). RMS errors show similar trends with an error of 0.15 in MA and of 1.10 mm in position. Therefore, we conclude that we were able to demonstrate the feasibility of an sEMG-assisted inverse modelling algorithm for the perioral region. This approach may help to solve the ambiguity problem in inverse modelling and may be useful, for instance, in future applications for preoperatively predicting treatment-related function loss.

## Introduction

Biomechanical modelling aims to represent human body dynamics as accurately as possible with mathematical equations, simulating and evaluating human movement and motor control while estimating the resulting internal and external forces. This can be useful in preoperative decision making. For instance, in children with cerebral palsy, Lofterød *et al*. evaluated the effect of providing 3D gait analysis information on preoperative surgical planning, finding that in the majority of cases surgical planning had been modified to incorporate important gait analysis data^1^.

Similar models are urgently needed in the field of Head and Neck Surgery, as well. Modelling of the perioral region may improve treatment and counselling of head and neck cancer patients, particularly by assessing functional inoperability, when surgical resection of a tumour will lead to unwanted severe loss of function^2^, and other organ-sparing treatments should be considered instead, e.g. chemotherapy, radiotherapy, photodynamic therapy, or any combination thereof. Human estimation of post-surgical function loss is by nature subjective and unreliable^3^. Therefore, there is urgent clinical need for tools that can predict patient-specific function loss objectively and quantitatively^4–8^. Promising results have been obtained with patient-specific biomechanical models of the face^9–11^, oral cavity^12,13^ and tongue^4,14,15^, including models that can simulate pharyngeal bolus transport^13,16^. Adding patient-specific neural control to such models by means of surface EMG (sEMG)-assisted inverse dynamics will be an essential step forward, as this will provide insight into pathophysiological dynamics and potential compensatory mechanisms after virtual resection of specific muscles.

There are two main types of modelling dynamics in biomechanics. Forward modelling, or forward-dynamics simulation, is the process of controlling a biomechanical model with given (muscle) activation signals, calculating the resulting forces with the equations of motion to ultimately obtain the corresponding functional movement. Inverse modelling, or inverse-dynamics simulation, is the opposite process, estimating the underlying muscle activation signals from measured actual forces or movements by using a biomechanical model with a mathematical optimisation criterion.

Inverse problems in biomechanical modelling are often mathematically ill-posed because of muscle redundancy: similar functional movements can be performed by different sets of muscles. This so-called load-sharing problem^17,18^ poses a significant challenge: to predict a patient’s motor behaviour accurately, the simulations must “share” muscle activations in the same way the patient does.

Literature reports various strategies to tackle the load-sharing problem, but these generally apply to models of the arms or legs. A recent paper by Yamasaki *et al*. shows that higher-order derivatives in static optimisation and forward-inverse dynamics can improve the estimation of muscle activation in highly dynamic motions within a simple musculoskeletal model that includes a one-degree-of-freedom (1DOF) hinge joint^19^. Some authors enforced co-contraction of antagonistic muscles using 1DOF hinge joint models^20–22^ or multi-body models^23^, while others used energy-based load-sharing cost terms^24,25^. Hybrid models have combined forward and inverse modelling by using algorithms that can derive neural activation strategy information from the muscle activation signals obtained with EMG. Such so-called EMG-assisted, EMG-informed, EMG-calibrated, or EMG-tracking algorithms were successfully applied in biomechanical models of the trunk^26–28^, shoulder and arm^29^, and legs^30,31^. Another feat has been the creation of a toolbox for calibrated EMG-informed neuro-musculoskeletal modelling (CEINMS)^32^. Reports on inverse modelling of the perioral region are scarce^33–35^, and only few involve EMG measurements^36^.

This paper aims to establish an sEMG-assisted inverse-modelling method that can be applied to 3D lip movements. We hypothesise that the addition of sEMG will allow for realistic inverse modelling solutions incorporating patient-specific activation strategies. If true, an sEMG-based model will be able to show the immediate functional outcome of surgery and also, if patients prove unable to relearn their functions, the final outcome. The proposed method is an adaptation of the so-called tracking-based inverse controller in ArtiSynth created by Stavness *et al*.^15^. This paper has been organised as follows. Section 2 summarises the static optimisation algorithm and introduces our adaptations. Section 3 describes the acquisition of experimental data and the pre-processing required. Section 4 reports on the use of these data in three experiments conducted to test the algorithm. Section 5 contains the discussion. The paper ends with our conclusion.

## EMG-assisted static optimisation

Our EMG-assisted inverse modelling algorithm is based on the inverse tracking controller in ArtiSynth developed by Stavness *et al*.^15^. They used a combined movement target term and an *l* ^2^-norm regularisation term, which resulted in a quadratic programming problem. In the current paper, we stacked the position coordinates of a set of ten tracked 3D marker points on the lips in a 30*D* vector ${{\bf{z}}}_{t}(k)$ where $k$ is the discrete time index. For brevity, we shall use the notation ${{\bf{z}}}_{t}$ instead of ${{\bf{z}}}_{t}(k)$. The model-predicted positions **z**(*k*) depend on ${\bf{a}}(k-1)$, which is the vector of muscle activations at time $k-1$, and on the previous state ${\bf{z}}(k-1)$. This is denoted by ${\bf{z}}(k)={{\bf{f}}}_{{\rm{m}}}({\bf{a}}(k-1),{\bf{z}}(k-1))$, where **f**_m_() is a state-space description representing the biomechanical model. For brevity, we shall write **a** instead of ${\bf{a}}(k-1)$ and **f**_m_(**a**) instead of ${{\bf{f}}}_{{\rm{m}}}({\bf{a}}(k-1),{\bf{z}}(k-1))$. Note also that the elements of **a** are limited to the interval [0,1]. The technology of sEMG provides indirect measurements of the innervation of each muscle. These measurements provide quantitative indications of the activations and are therefore denoted by **a**_*t*_, which gives rise to the following quadratic cost function:

1 $$J({\bf{a}})=\frac{1}{2}{({{\bf{f}}}_{{\rm{m}}}({\bf{a}})-{{\bf{z}}}_{t})}^{{\rm{T}}}{\bf{M}}({{\bf{f}}}_{{\rm{m}}}({\bf{a}})-{{\bf{z}}}_{t})+\frac{1}{2}{{\bf{a}}}^{{\rm{T}}}{\bf{Aa}}+\frac{1}{2}{({\bf{a}}-{{\bf{a}}}_{prev})}^{{\rm{T}}}{\bf{D}}({\bf{a}}-{{\bf{a}}}_{prev})+\frac{1}{2}{({\bf{a}}-{{\bf{a}}}_{t})}^{{\rm{T}}}{\bf{E}}({\bf{a}}-{{\bf{a}}}_{t})$$

With ${{\bf{a}}}_{prev}={\bf{a}}(k-2)$. The matrices **M**, **A**, **D**, and **E** are matrices that weigh different cost aspects. The term with **M** assures that model positions are close to measured positions. The term with **A** is a regulation term to tame the found activation signals. The term with **D** prevents large fluctuations of the found activations. Finally, the term with **E** assures that the estimated activations are consistent with the measured sEMG signals. In our experiments, the numerical values of the matrices were as follows: ${\bf{M}}=diag(1)$, ${\bf{A}}=diag(0.05)$, ${\bf{D}}=diag(0.005)$, and ${\bf{E}}=diag(em{g}_{val})$ or ${\bf{E}}=diag(0)$ in case inverse modelling is performed without sEMG tracking. *emg*_*val*_ was determined during the experiments.

To minimise the cost function in equation (1), the expression was worked out to a form:

2 $$\hat{{\bf{a}}}=\mathop{\text{arg}\,\min }\limits_{{\bf{a}}\in [0,1]}\{\frac{1}{2}{{\bf{a}}}^{{\rm{T}}}{{\bf{H}}}^{{\rm{T}}}{\bf{Ha}}-{{\bf{a}}}^{{\rm{T}}}{{\bf{H}}}^{{\rm{T}}}{\bf{b}}\}$$

in which irrelevant terms in equation (1) were dropped, and a linearised approximation of the state-space model was used based on Taylor series expansion. Equation (2) is recognised as a quadratic programming problem for which stable, numerical solutions are available. The seed for the inversion was always set to the estimated muscle activity of the previous frame. The initial frame’s seed was always set to zero muscle activity.

## Data acquisition and pre-processing

### Volunteers and data acquisition

**Figure 1:**
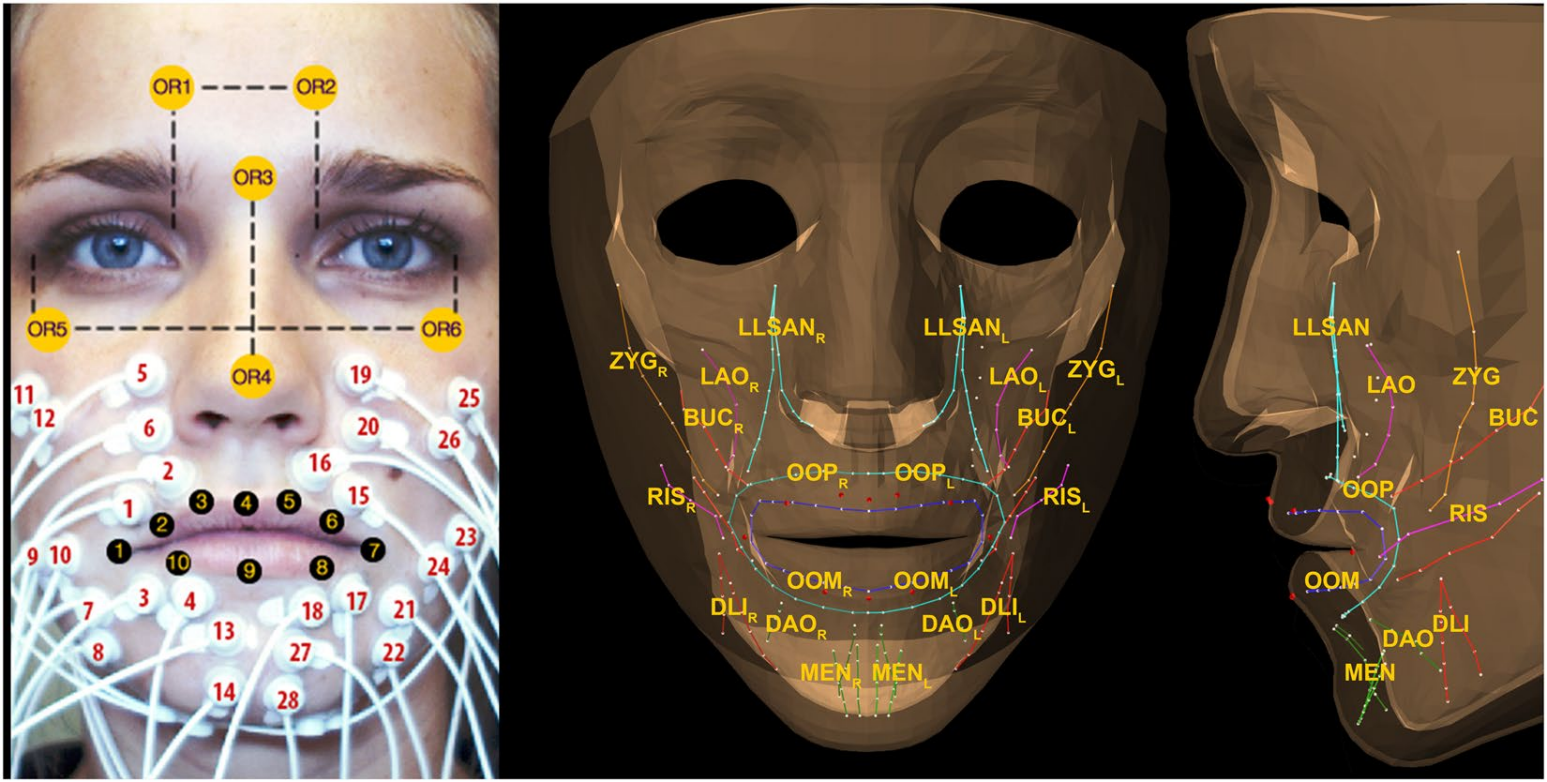
Left: Surface electrode locations, orientation markers, and lip markers. Right: Anterior-posterior view and lateral view of the model and the model**’**s muscle bundles and lip markers. The muscles are abbreviated as follows: zygomaticus major (ZYG), risorius (RIS), levator labii superioris alaeque nasi (LLSAN), levator anguli oris (LAO), buccinator (BUC), orbicularis oris peripheralis (OOP) and marginalis (OOM), depressor labii inferior (DLI), depressor anguli oris (DAO), and mentalis (MEN). Subscript _L_ is for left side and subscript _R_ for right side. Adopted from Eskes *et al*.^7^.

To perform inverse modelling experiments, we used data of six healthy volunteers (three males, three females), with a mean age of 25 years (range 21 to 30 years), whom we had recruited for our previous studies^6,7^. For details, see Eskes *et al*.^6^. Essentials are summarised below. The data are available on the Open Science Framework^37^. Written consent was obtained for publishing the photograph in Fig. 1.

sEMG signals *s*_*m *_(*m* muscle channels) were recorded with the TMSi® Porti™ system (TMSi®, Oldenzaal, the Netherlands) and micro-sEMG electrodes (1.5 mm diameter, Ag/AgCl, disc-shaped, with actively shielded cables). The following muscles were measured in bipolar configuration according to the optimal placement described by Lapatki *et al*.^38^: the Orbicularis Oris Superior (OOS), the Orbicularis Oris Inferior (OOI), the Depressor Anguli Oris (DAO), the Mentalis (MEN), the Risorius (RIS), the Zygomaticus major (ZYG), and the Levator Labii Superioris Alaeque Nasi (LLSAN) (Fig. 1). The sEMG signals were bandpass filtered with high-pass and low-pass cut-off frequencies of 15 and 500 Hz, respectively.

To acquire 3D lip movements, we tracked six optical face markers $({{\rm{X}}}_{{OR}}\in {{\mathbb{R}}}^{18})$ for head orientation and ten optical lip markers $({\rm{X}}\in {{\mathbb{R}}}^{30})$ at 100 frames per second using a triple camera set-up (avA1000–100gc, Basler AG, Ahrensburg, Germany), which we had developed for assessing tongue mobility and capturing tongue movement after hypoglossal nerve stimulation^8,39^ (Fig. 1).

**Figure 2:**
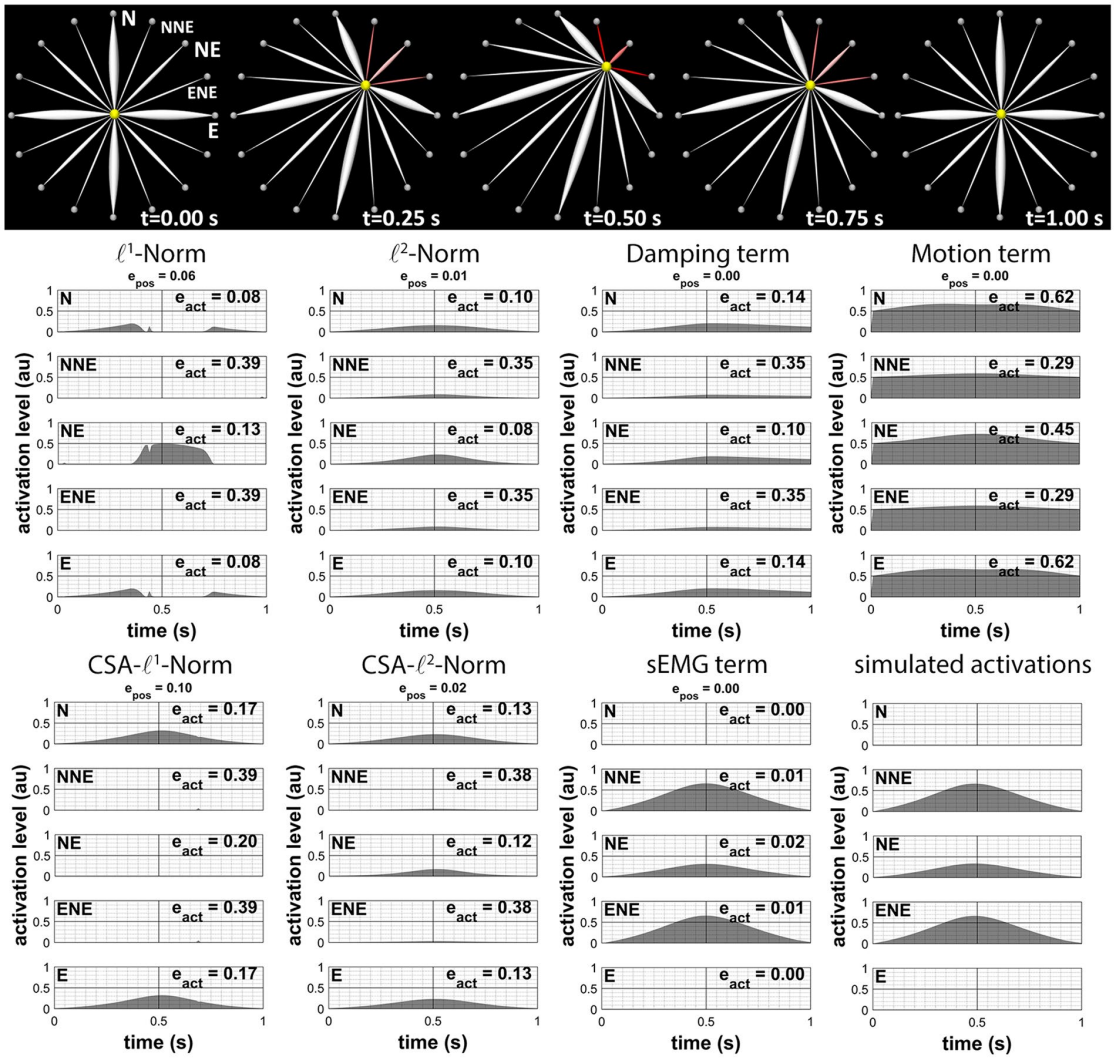
The top row shows the point-mass model with simulated forward movement to the northeast corner. The muscles’ red colour intensities illustrate the extent of activation. The eight graphs below show the influence of the different cost terms on the estimated muscle activations of five muscles during inverse modelling. The original simulated activations are given in the lower right corner (‘au’ is for ‘arbitrary units’).

We asked the volunteers to perform six different instructions once: A. purse lips, B. raise upper lip, C. depress mouth corners, D. voluntary smile, E. draw mouth corner to the left, then to the right, and again to the left, and F. purse lips to closed-mouth smile to purse lips (Eskes *et al*.^7^, Fig. 2).

The experiments were approved by the Medical Research Ethics Committee of the Netherlands Cancer Institute and all volunteers gave written informed consent.

### Finite-element face model

We performed inverse modelling on the generic face model used in Eskes *et al*.^7^ (Fig. 1), which was based on the work performed at ICP/GIPSA and TIMC-IMAG laboratories in Grenoble^40,41^, with details published by Nazari *et al*.^42^. Their ANSYS® model was ported to ArtiSynth and was named the reference face model^43–45^. With soft tissues represented in three layers of elements, this model had 6342 elements (6024 linear hexahedral and 318 linear wedge) and 8720 nodes. Fourteen muscle groups were available as muscle fibres. We created finite-element muscles, which were defined as the elements surrounding the muscle fibres within a radius of 5 mm. The elements of the Orbicularis Oris muscles were manually assigned. All these muscle elements were given muscle properties as described by Blemker *et al*.^46^. The bony parts, the mandible and maxilla, were modelled as rigid bodies. We used literature-based common muscle model parameters for all volunteers^7,11^, with the exception of maximum muscle stress (σ_max_). We optimised the stress parameter per volunteer starting at 300 kPa and gradually decreased σ_max_ repeatedly with 10 percent until the simulation ran smoothly without creating inverted elements. Simulations were performed on two workstations with intel Xeon core and one laptop computer with an intel i7 core.

### sEMG to normalised model activations

The model used Orbicularis Oris Peripheralis (OOP) and Marginalis (OOM) definitions. Therefore, these activations were constructed from the measured OOS and OOI activations, taking into account the information about activation patterns described by Flynn *et al*.^11^. The Buccinator (BUC), the Depressor Labii Inferior (DLI), and the Levator Anguli Oris (LAO) were not directly measured but derived from the measured muscles as follows:

3 $${s}_{OOP}=0.50\,({s}_{OOS}+{s}_{OOI})$$

4 $${s}_{OOM}=0.10({s}_{OOP}+{s}_{OOI})$$

5 $${s}_{BUC}=0.50({s}_{RIS}+{s}_{ZYG})$$

6 $${s}_{LAO}=0.75{s}_{LSSAN}$$

7 $${s}_{DLI}=0.75{s}_{DAO}$$

For the different instructions, the following muscles were considered relevant^7,11^:

A. OOP, OOM, and BUC
B. LLSAN
C. DAO and MEN
D. ZYG, RIS, LAO, LLSAN, DAO, and DLI
E. OOP, OOM, LLSAN, RIS, ZYG, BUC, and LAO
F. OOP, OOM, LLSAN, RIS, ZYG, BUC, and LAO

In previous research, we found the following procedure to be optimal for transforming measured sEMG signals into normalised muscle activations^5–7^. We first calculated the Willison Amplitude with ${s}_{\mathrm{lim}}=10\,{\rm{mV}}$ over sliding windows of 200 ms with maximum overlap:

8 $${g}_{m}(t,i,r)=\sum _{n=1}^{N-1}[\,f(|{s}_{m}(t+n-1)-{s}_{m}(t+n)|)]$$

$$\begin{array}{cc}{\rm{with}} & f({s}_{m})=\{\begin{array}{cc}\begin{array}{c}1\\ 0\end{array} & \begin{array}{c}\begin{array}{cc}{\rm{if}} & {s}_{m}\ge {s}_{\mathrm{lim}}\end{array}\\ {\rm{otherwise}}\end{array}\end{array}\end{array}\begin{array}{l}i\,{\rm{is}}\,{\rm{instruction}}\,{\rm{index}}\\ r\,{\rm{is}}\,{\rm{repetition}}\,{\rm{index}}\end{array}$$

The feature *g*_*m*_(*t*, *i*, *r*) was calculated from the measured sEMG *s*_*m*_(*t*) of muscle *m*, where *t* was the time index of the EMG signals, and *n* the running time index within each sliding window consisting of *N* samples. This was done for all instructions *i* and repetitions *r* (in this case $r=1$). The feature *g*_*m*_(*t*, *i*, *r*) was normalised according to:

9 $${g}_{norm,m}(t,i,r)=\frac{{g}_{m}(t,i,r)-\mathop{\min }\limits_{t}({g}_{m}(t,i,r))}{\mathop{\max }\limits_{t}({g}_{m}(t,i,r))-\mathop{\min }\limits_{t}({g}_{m}(t,i,r))}$$

### Registration of measured 3D lip markers to generic face model

As each face has unique dimensions, we had to apply a registration to allow for movement tracking and root mean square (RMS) error comparison of the generic face model’s lip markers with the measured lip markers. We registered each measured coordinate according to equation (10):

10 $${Z}_{d}^{norm}(k)=({Z}_{d}(k)-{\mu }_{d}^{Z})(\frac{{\sigma }_{d}^{X}}{{\sigma }_{d}^{Z}})+{\mu }_{d}^{X}$$

*Z*_*d*_(*k*) is the $d \mbox{-} th$ element from the original measured position vector **z**_*t*_(*k*). The normalised measured positions are denoted by ${{\rm{Z}}}_{d}^{norm}(k)$. In equation (10), ${\mu }_{d}^{Z}$ is the mean of the measured position coordinates, and ${\mu }_{d}^{X}$ the mean of the model’s position coordinates. The standard deviation of the model’s position coordinates is denoted by ${\sigma }_{d}^{X}$, whereas the standard deviation of the measured position coordinates is given by ${\sigma }_{d}^{Z}$.

### Performance measures

To perform quantitative evaluation, we used the RMS error, *e*_*pos*_, that was calculated over time and over the markers via:

11 $${e}_{pos}=\sqrt{\frac{{\sum }_{k=1}^{K}({\sum }_{d=1}^{D}{({Z}_{d}^{norm}(k)-{Z}_{d}(k))}^{2})}{KD}}\cdot \sqrt{3}$$

With *k* being the discrete time index, *K* the number of time samples, and *Z*_*d*_(*k*) the model’s lip marker position coordinates. $D=30$ reflects the dimensions, i.e. 10 markers with 3 coordinates each. The factor $\sqrt{3}$ was introduced because we wanted to express the RMS in terms of distances, rather than in terms of coordinates.

The 3D correlation coefficients were calculated as described by Pitermann *et al*.^36^. The mean position **μ**_*z*_ of a 3D lip marker trajectory, with samples ${{\bf{Z}}}_{t}=({x}_{t},{y}_{t},{v}_{t})$, was calculated with equation (12):

12 $${{\boldsymbol{\mu }}}_{Z}=(\frac{1}{T}\sum _{t=1}^{T}{x}_{t},\frac{1}{T}\sum _{t=1}^{T}{y}_{t},\frac{1}{T}\sum _{t=1}^{T}{v}_{t})$$

The standard deviation **σ**_*z*_ of **Z**_*t*_ was calculated with equation (13):

13 $${{\boldsymbol{\sigma }}}_{Z}=\sqrt{\frac{1}{T-1}\sum _{t=1}^{T}{\Vert {{\bf{Z}}}_{t}-{{\boldsymbol{\mu }}}_{Z}\Vert }^{2}}$$

The 3D correlation coefficient *ρ*_3*D*_ between 3D landmark trajectories **Z**_*t*_ and **X**_*t*_ was calculated with equation (14):

14 $${\rho }_{3D}=\frac{\frac{1}{T}\sum _{t=1}^{T}{{{\bf{Z}}}_{t}}^{T}{{\bf{X}}}_{t}-{{{\boldsymbol{\mu }}}_{Z}}^{T}{{\boldsymbol{\mu }}}_{X}}{{{\boldsymbol{\sigma }}}_{Z}{{\boldsymbol{\sigma }}}_{X}}$$

The RMS error was also calculated for the activations (*e*_*act*_) according to equation (15) with **g**(*t*) being the normalised feature values and **a**(*t*) the inverse calculated activation values, whereas Pearson’s correlation coefficient was used as an activation correlation measure.

15 $${e}_{act}=\sqrt{\frac{{\sum }_{t=1}^{T}{({\bf{g}}(t)-{\bf{a}}(t))}^{2}}{T}}$$

For all experiments, we compared the inverse calculated activation signals with the original sEMG features using the RMS error and Pearson’s correlation coefficient. Also, the movement tracking errors (*e*_*pos*_ and *ρ*_3*D*_) were calculated for all experiments. Together, these measures give an indication of performance.

## Experiments

In this study, we performed three different experiments to investigate the added value of sEMG-assisted inverse modelling:

I. A simple muscle contraction to test feasibility of the model and implementation of the inverse methods
II. Inverse simulations with synthetic data produced by the sEMG-driven forward model. Inverse modelling was guided by 3 different sEMG constraints: no constraint, using all muscles (*act*_*all*_), and using relevant muscles (*act*_*rel*_). By comparing the results of these three constraints, we could test our method for feasibility inside the mathematical universe of the face model.
III. Inverse simulations with measurement data containing 3D position data of ten lip markers and sEMG data of fourteen facial muscles. This experiment was conducted to assess the contribution of sEMG in a realistic situation.

### Experiment I: Test Cost term implementation by means of a simple point-mass system

#### Goal and experimental set-up

To test our implementation of the cost function, we first created a simulated muscle activation pattern, contracting the north-north-east, north-east, and east-north-east muscle bundles of the point-mass system as shown in Fig. 2 ^47^. It should be noted that the muscles have different maximum isometric forces, the thick muscles being more powerful than the thinner muscles. Next, inverse modelling was performed, first alternating the cost terms and finally using all cost terms at once. We expected to find that IM with each cost-term alone would not result in calculated IM activations that were similar to the simulated activation patterns, except for IM with the sEMG term, which would probably mimic the forward simulation. When using all cost terms together, we expected there would be a trade-off between the different cost terms, which would likely cause a result that was less perfect but more usable in the real application. In line with logic, when testing a cost term alone, we set its weighing factor at one. When testing all cost-terms together, we set the various weighing factors as described in section 2: ${\bf{M}}=diag(1)$, ${\bf{A}}=diag(0.05)$, ${\bf{D}}=diag(0.005)$, and ${\bf{E}}=diag(em{g}_{val})$ with in this case $em{g}_{val}=0.05$.

#### Results

**Figure 3:**
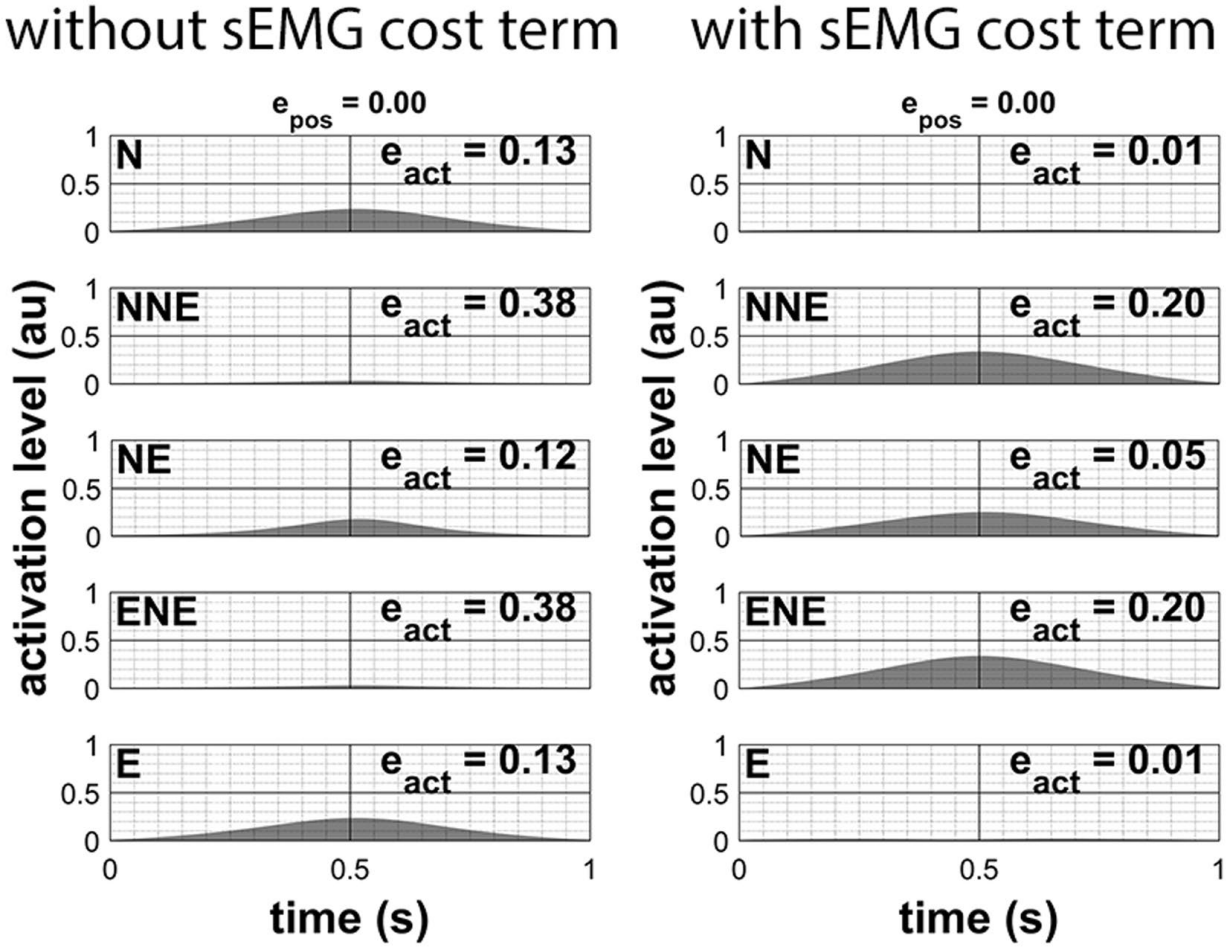
Inverse modelling with all cost terms active except for the sEMG term. Left: the estimated activations when not using the sEMG term. Right: estimated activations when using all cost terms including the sEMG term (‘au’ is for ‘arbitrary units’).

For the point-mass system, movement tracking errors were similar in all simulations, whereas activation patterns differed greatly. Using the motion term alone produced a very stiff system, whereas the *l* ^2^-norm distributed the forces over the different muscles in the same way the damping term did. Including only the sEMG term showed minimal differences between the inverse calculated activation and the simulated activation and resulted in a good forward solution (*e*_*pos*_). When using all cost terms together, including our sEMG term, we found that muscle activation patterns were still good (Fig. 3) while used muscle activation strategies improved considerably over performance with individual cost terms or all cost terms combined with exclusion of the sEMG term. However, it should be noted that the solution depends on the weighing factors of the cost terms, e.g. when too much sEMG information is used, the result will mimic the forward solution.

The results were not perfect because of the other cost terms in the objective function and because of integration, which adds noise. Even when we activated only the sEMG target term, there was still a small error between the inverse calculated activations and the simulated sEMG pattern used in forward modelling. Larger errors occurred when we applied all cost terms in the inverse modelling of the point-mass model, which is a direct consequence of taking into account all cost terms, where the sum of all terms should be small, instead of only minimising the sEMG term.

#### Conclusion

To conclude, these experiments justified our approach and showed that sacrificing only a little performance in movement tracking resulted in major improvement in muscle activation tracking. Neither the use of any original cost term by itself nor any combined use of cost terms resulted in the correct muscle activation strategy. Incorporation of the sEMG cost term greatly improved the estimated muscle activations while keeping movement tracking orders in the same range. The weighing factors influence the result and should be determined experimentally for the next experiments.

### Experiment II: Inverse modelling using simulated data

#### Goal and experimental set-up

**Figure 4:**
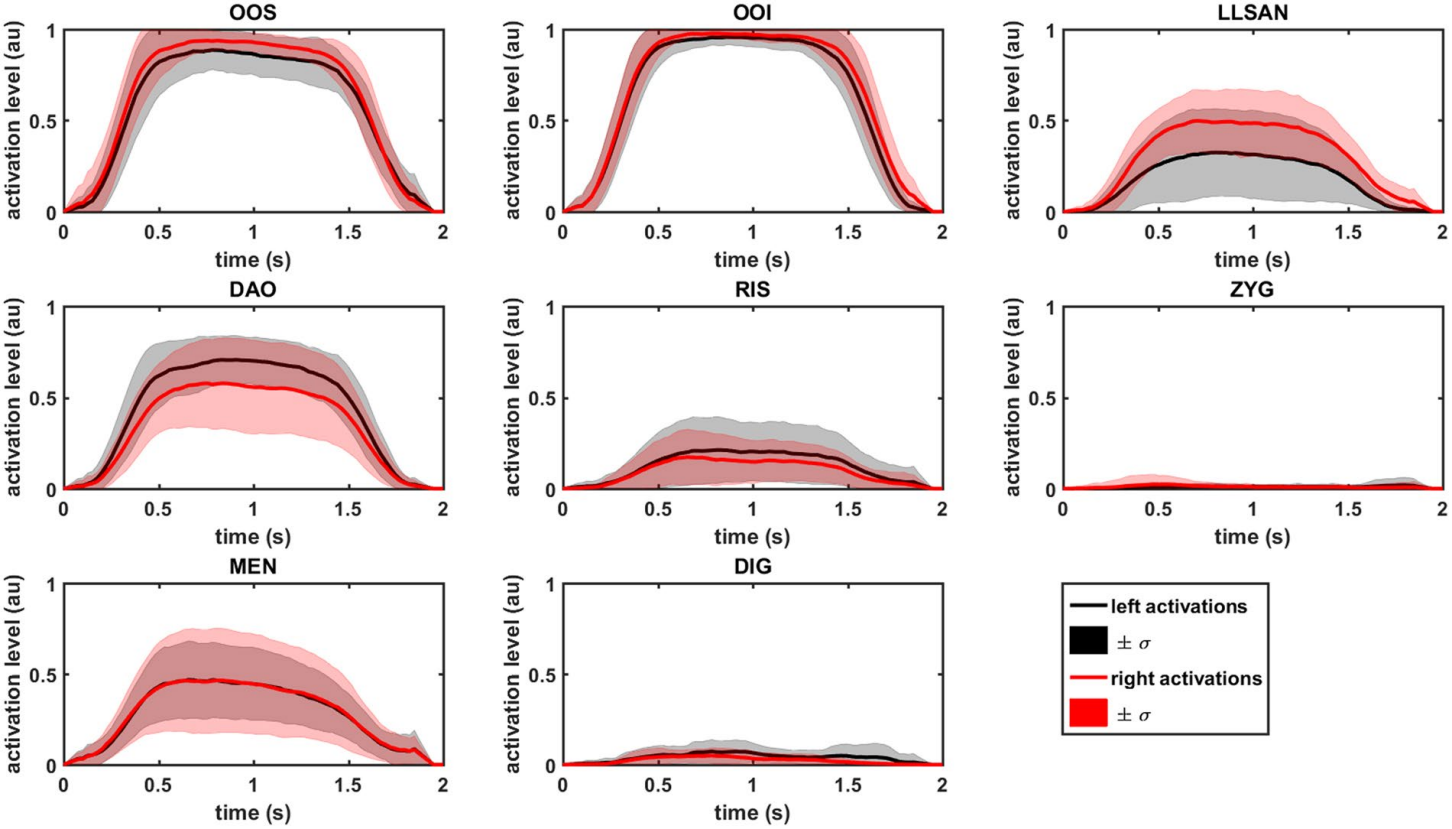
Muscle activation patterns calculated from sEMG features of the instruction ‘purse lips’ as input for forward modelling. The mean and standard deviations of all volunteers are shown for all measured muscles. High standard deviations show the volunteer-specific activations, with asymmetry in the DAO and LLSAN muscles, in particular. (‘au’ is for ‘arbitrary units’).

To test the inverse modelling approach within the mathematical universe of the face and assess its feasibility, we started with a standard inverse-modelling approach^15^. To first evaluate this approach in a simple situation, we used our forward-modelling results as motion targets for this experiment^7^. After activating the relevant muscles per instruction (*act*_*rel*_), the forward simulation produced 3D trajectory data of the lip markers. Since this movement lies within the range of the model (position, acceleration) there is no need for registration, which could induce error, and the movement can function as a first indicator of feasibility. Figure 4 depicts the mean activations and their standard deviations based on all volunteers for the measured muscles. For use as input for the forward model, they were adjusted with equations (3) to (7). In this experiment, we used three constraints for the IM sEMG term: no sEMG, including all muscle activations (*act*_*all*_), and including relevant muscle activations (*act*_*rel*_). Thus, the sEMG term’s penalty matrix **E** was set to zero if no activation targets were used, while we experimentally obtained the optimal value using three different values for *emg*_*val*_ to get an idea of the influence of the sEMG term: $5\times {10}^{-5}$, $5\times {10}^{-4}$, and $5\times {10}^{-3}$. Now, a trade-off between muscle activation tracking and movement tracking will be made. In this experiment, all muscles were used (*act*_*all*_). After obtaining the optimal *emg*_*val*_, the constraints *act*_*all*_ and *act*_*rel*_ were tested.

#### Results

**Figure 5:**
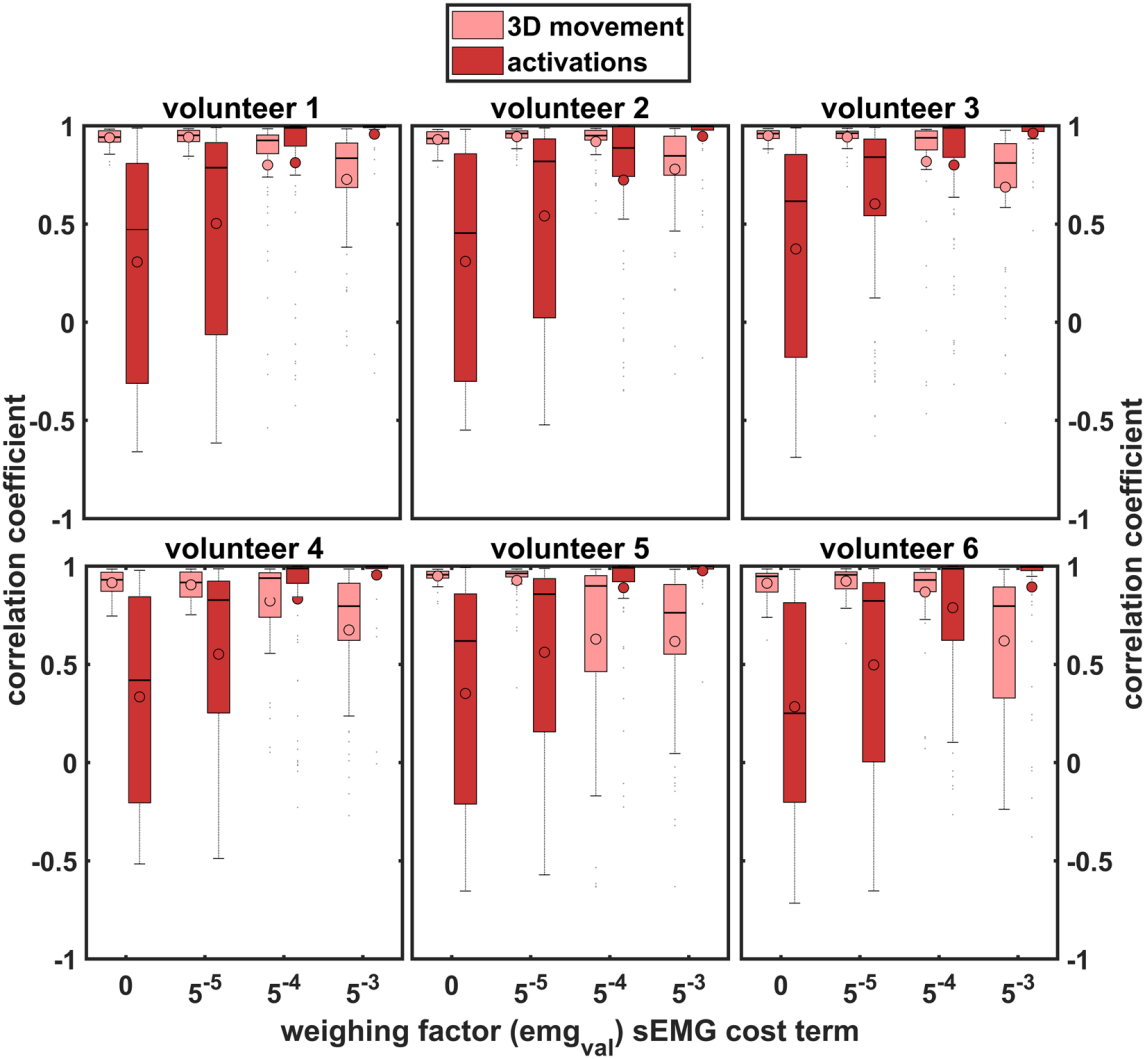
Influence of the sEMG cost weighing factor on the 3D correlation coefficients of movement and on Pearson’s correlation coefficients of calculated muscle activations and sEMG features. The median is shown with a horizontal line and the mean with a dot. The boxes give first to third quartiles and the outer horizontal lines reflect minimum and maximum values.

The influence of the sEMG cost term and thus the optimal weighing factor can be derived from Fig. 5. All volunteers show the same pattern: a weighing factor of $5\times {10}^{-3}$ actually results in forward modelling as it depends too much on the muscle activations patterns, whereas $5\times {10}^{-4}$ appears to be the optimal value of all tested factors.

Table 1 gives the RMS error between the target lip markers and the models’ lip markers *e*_*pos*_ averaged over all instructions and volunteers for experiments II and III, as well as the *e*_*act*_ between the models’ calculated activations and measured muscle activations. Similarly, Table 2 shows the 3D correlation coefficients *ρ*_3*D*_ between model markers and measurement markers and Pearson’s correlation coefficients *ρ* between calculated model activations and measured muscle activations.

**Table 1:** Root mean square errors. The mean (µ) and standard deviation (σ) of the *e*_*pos*_ for the ten lip markers and *e*_*act*_ for the ten muscles left and right over all volunteers and all instructions.

Marker # Muscle	1 OOM	2 OOP	3 LLSAN	4 DAO	5 RIS	6 ZYG	7 MEN	8 BUC	9 DLI	10 LAO	Average
EXPERIMENT II Without sEMG	**µ (σ)**	**µ (σ)**	**µ (σ)**	**µ (σ)**	**µ (σ)**	**µ (σ)**	**µ (σ)**	**µ (σ)**	**µ (σ)**	**µ (σ)**	**µ (σ)**
Lip markers	0.84 (0.35)	0.34 (0.13)	0.28 (0.11)	0.27 (0.13)	0.29 (0.11)	0.36 (0.14)	0.89 (0.40)	0.40 (0.23)	0.41 (0.35)	0.40 (0.23)	**0**.**45 (0**.**35)**
Left muscles	0.08 (0.05)	0.32 (0.18)	0.22 (0.13)	0.36 (0.23)	0.16 (0.14)	0.14 (0.14)	0.37 (0.22)	0.12 (0.10)	0.27 (0.16)	0.28 (0.15)	**0**.**23 (0**.**15)**
Right muscles	0.08 (0.05)	0.31 (0.17)	0.19 (0.12)	0.42 (0.23)	0.16 (0.14)	0.14 (0.15)	0.36 (0.20)	0.12 (0.11)	0.31 (0.16)	0.26 (0.16)	**0**.**23 (0**.**15)**
With sEMG *act*_*all*_	**µ (σ)**	**µ (σ)**	**µ (σ)**	**µ (σ)**	**µ (σ)**	**µ (σ)**	**µ (σ)**	**µ (σ)**	**µ (σ)**	**µ (σ)**	**µ (σ)**
Lip markers	0.81 (0.35)	0.31 (0.10)	0.29 (0.09)	0.29 (0.11)	0.30 (0.09)	0.33 (0.10)	0.84 (0.39)	0.39 (0.20)	0.46 (0.32)	0.39 (0.21)	**0**.**44 (0**.**20)**
Left muscles	0.10 (0.07)	0.35 (0.18)	0.19 (0.13)	0.29 (0.21)	0.14 (0.14)	0.10 (0.10)	0.33 (0.23)	0.08 (0.07)	0.25 (0.19)	0.31 (0.16)	**0**.**21 (0**.**15)**
Right muscles	0.10 (0.07)	0.33 (0.18)	0.16 (0.12)	0.32 (0.19)	0.14 (0.14)	0.11 (0.10)	0.32 (0.21)	0.09 (0.08)	0.30 (0.18)	0.26 (0.18)	**0**.**21 (0**.**15)**
With sEMG *act*_*rel*_	**µ (σ)**	**µ (σ)**	**µ (σ)**	**µ (σ)**	**µ (σ)**	**µ (σ)**	**µ (σ)**	**µ (σ)**	**µ (σ)**	**µ (σ)**	**µ (σ)**
Lip markers	1.07 (0.55)	0.50 (0.25)	0.50 (0.27)	0.51 (0.27)	0.51 (0.27)	0.53 (0.26)	1.11 (0.57)	0.62 (0.33)	0.65 (0.34)	0.63 (0.36)	**0**.**66 (0**.**55)**
Left muscles	0.27 (0.30)	0.10 (0.10)	0.08 (0.06)	0.04 (0.06)	0.06 (0.06)	0.05 (0.06)	0.02 (0.04)	0.20 (0.19)	0.02 (0.05)	0.16 (0.15)	**0**.**10 (0**.**11)**
Right muscles	0.27 (0.28)	0.10 (0.09)	0.08 (0.07)	0.04 (0.06)	0.06 (0.07)	0.06 (0.07)	0.02 (0.04)	0.19 (0.19)	0.03 (0.05)	0.12 (0.14)	**0**.**10 (0**.**11)**
EXPERIMENT III Without sEMG	**µ (σ)**	**µ (σ)**	**µ (σ)**	**µ (σ)**	**µ (σ)**	**µ (σ)**	**µ (σ)**	**µ (σ)**	**µ (σ)**	**µ (σ)**	**µ (σ)**
Lip markers	1.83 (1.61)	1.32 (0.79)	1.26 (0.78)	1.20 (0.77)	1.21 (0.81)	1.45 (1.00)	1.91 (1.56)	1.24 (0.98)	1.23 (0.91)	1.32 (1.17)	**1**.**40 (1**.**61)**
Left muscles	0.16 (0.19)	0.33 (0.19)	0.30 (0.17)	0.48 (0.22)	0.22 (0.16)	0.15 (0.16)	0.37 (0.23)	0.19 (0.14)	0.33 (0.16)	0.29 (0.15)	**0**.**28 (0**.**18)**
Right muscles	0.12 (0.15)	0.34 (0.19)	0.30 (0.14)	0.42 (0.22)	0.24 (0.20)	0.17 (0.18)	0.37 (0.23)	0.20 (0.17)	0.30 (0.16)	0.30 (0.16)	**0**.**28 (0**.**18)**
With sEMG *act*_*all*_	**µ (σ)**	**µ (σ)**	**µ (σ)**	**µ (σ)**	**µ (σ)**	**µ (σ)**	**µ (σ)**	**µ (σ)**	**µ (σ)**	**µ (σ)**	**µ (σ)**
Lip markers	1.33 (0.66)	1.05 (0.46)	1.02 (0.49)	1.02 (0.44)	1.03 (0.48)	1.10 (0.53)	1.52 (0.77)	0.97 (0.62)	1.01 (0.74)	0.95 (0.51)	**1**.**10 (0**.**66)**
Left muscles	0.08 (0.06)	0.20 (0.16)	0.09 (0.06)	0.07 (0.03)	0.07 (0.04)	0.02 (0.02)	0.10 (0.11)	0.23 (0.18)	0.31 (0.14)	0.31 (0.15)	**0**.**15 (0**.**10)**
Right muscles	0.06 (0.03)	0.21 (0.16)	0.08 (0.07)	0.06 (0.03)	0.07 (0.04)	0.02 (0.02)	0.10 (0.11)	0.21 (0.15)	0.28 (0.15)	0.29 (0.15)	**0**.**14 (0**.**09)**

**Table 2:** 3D and 2D correlations. The mean (µ) and standard deviations (σ) of the 3D correlations for the ten lip markers and the Pearson’s correlation coefficients of the facial muscles bilaterally overall for all volunteers and all instructions.

Marker # Muscle	1 OOM	2 OOP	3 LLSAN	4 DAO	5 RIS	6 ZYG	7 MEN	8 BUC	9 DLI	10 LAO	Average
EXPERIMENT II Without sEMG	**µ (σ) [ρ]**	**µ (σ) [ρ]**	**µ (σ) [ρ]**	**µ (σ) [ρ]**	**µ (σ) [ρ]**	**µ (σ) [ρ]**	**µ (σ) [ρ]**	**µ (σ) [ρ]**	**µ (σ) [ρ]**	**µ (σ) [ρ]**	**µ (σ) [ρ]**
Lip markers	0.92 (0.07)	0.94 (0.05)	0.94 (0.05)	0.94 (0.05)	0.94 (0.04)	0.93 (0.04)	0.92 (0.06)	0.93 (0.04)	0.94 (0.05)	0.93 (0.05)	**0**.**93 (0**.**07)**
Left muscles	0.41 (0.47)	0.46 (0.41)	0.50 (0.52)	−0.19 (0.48)	0.37 (0.60)	0.52 (0.51)	0.29 (0.46)	0.38 (0.57)	−0.07 (0.47)	0.02 (0.51)	**0**.**27 (0**.**50)**
Right muscles	0.42 (0.49)	0.41 (0.44)	0.52 (0.48)	−0.20 (0.50)	0.31 (0.59)	0.44 (0.53)	0.32 (0.46)	0.39 (0.55)	−0.09 (0.47)	0.11 (0.55)	**0**.**26 (0**.**51)**
With sEMG *act*_*all*_	**µ (σ) [ρ]**	**µ (σ) [ρ]**	**µ (σ) [ρ]**	**µ (σ) [ρ]**	**µ (σ) [ρ]**	**µ (σ) [ρ]**	**µ (σ) [ρ]**	**µ (σ) [ρ]**	**µ (σ) [ρ]**	**µ (σ) [ρ]**	**µ (σ) [ρ]**
Lip markers	0.94 (0.05)	0.94 (0.05)	0.93 (0.07)	0.92 (0.07)	0.93 (0.05)	0.94 (0.05)	0.94 (0.04)	0.94 (0.05)	0.90 (0.13)	0.93 (0.06)	**0**.**93 (0**.**05)**
Left muscles	0.39 (0.44)	0.49 (0.39)	0.64 (0.53)	0.57 (0.48)	0.45 (0.60)	0.59 (0.52)	0.59 (0.47)	0.41 (0.57)	0.19 (0.47)	0.00 (0.53)	**0**.**43 (0**.**50)**
Right muscles	0.40 (0.45)	0.44 (0.41)	0.68 (0.46)	0.63 (0.38)	0.40 (0.60)	0.51 (0.53)	0.60 (0.43)	0.39 (0.57)	0.16 (0.47)	0.16 (0.57)	**0**.**44 (0**.**49)**
With sEMG *act*_*rel*_	**µ (σ) [ρ]**	**µ (σ) [ρ]**	**µ (σ) [ρ]**	**µ (σ) [ρ]**	**µ (σ) [ρ]**	**µ (σ) [ρ]**	**µ (σ) [ρ]**	**µ (σ) [ρ]**	**µ (σ) [ρ]**	**µ (σ) [ρ]**	**µ (σ) [ρ]**
Lip markers	0.93 (0.06)	0.94 (0.04)	0.91 (0.09)	0.90 (0.11)	0.90 (0.12)	0.93 (0.05)	0.93 (0.06)	0.93 (0.05)	0.89 (0.12)	0.92 (0.05)	**0**.**92 (0**.**06)**
Left muscles	0.85 (0.11)	0.85 (0.11)	0.91 (0.07)	0.96 (0.02)	0.95 (0.04)	0.96 (0.04)	0.93 (0.08)	0.56 (0.45)	0.90 (0.10)	0.29 (0.49)	**0**.**82 (0**.**15)**
Right muscles	0.85 (0.10)	0.86 (0.10)	0.90 (0.08)	0.96 (0.01)	0.92 (0.06)	0.93 (0.07)	0.93 (0.07)	0.60 (0.39)	0.89 (0.09)	0.60 (0.22)	**0**.**84 (0**.**12)**
EXPERIMENT III Without sEMG	**µ (σ) [ρ]**	**µ (σ) [ρ]**	**µ (σ) [ρ]**	**µ (σ) [ρ]**	**µ (σ) [ρ]**	**µ (σ) [ρ]**	**µ (σ) [ρ]**	**µ (σ) [ρ]**	**µ (σ) [ρ]**	**µ (σ) [ρ]**	**µ (σ) [ρ]**
Lip markers	0.74 (0.23)	0.68 (0.25)	0.65 (0.21)	0.58 (0.24)	0.61 (0.23)	0.70 (0.23)	0.80 (0.16)	0.65 (0.35)	0.64 (0.26)	0.68 (0.31)	**0**.**67 (0**.**23)**
Left muscles	0.05 (0.47)	0.20 (0.55)	0.50 (0.41)	−0.11 (0.52)	0.22 (0.64)	0.42 (0.57)	0.00 (0.50)	0.40 (0.56)	0.05 (0.49)	0.15 (0.55)	**0**.**19 (0**.**53)**
Right muscles	0.05 (0.43)	0.20 (0.52)	0.43 (0.51)	−0.10 (0.48)	0.18 (0.60)	0.53 (0.51)	−0.04 (0.53)	0.40 (0.52)	0.09 (0.49)	0.33 (0.44)	**0**.**21 (0**.**50)**
With sEMG *act*_*all*_	**µ (σ) [ρ]**	**µ (σ) [ρ]**	**µ (σ) [ρ]**	**µ (σ) [ρ]**	**µ (σ) [ρ]**	**µ (σ) [ρ]**	**µ (σ) [ρ]**	**µ (σ) [ρ]**	**µ (σ) [ρ]**	**µ (σ) [ρ]**	**µ (σ) [ρ]**
Lip markers	0.77 (0.21)	0.74 (0.17)	0.65 (0.19)	0.62 (0.26)	0.67 (0.21)	0.75 (0.19)	0.73 (0.25)	0.68 (0.36)	0.66 (0.32)	0.69 (0.34)	**0**.**70 (0**.**21)**
Left muscles	−0.00 (0.49)	0.64 (0.43)	0.90 (0.28)	0.97 (0.09)	0.77 (0.41)	0.79 (0.38)	0.92 (0.21)	0.41 (0.51)	0.31 (0.47)	0.04 (0.52)	**0**.**57 (0**.**38)**
Right muscles	−0.00 (0.49)	0.63 (0.43)	0.91 (0.29)	0.94 (0.24)	0.79 (0.35)	0.82 (0.34)	0.89 (0.26)	0.39 (0.49)	0.33 (0.45)	0.29 (0.47)	**0**.**60 (0**.**38)**

As we evaluate these experiments, some comments have to be made. The experiments confirm the load-sharing problem: three different activation strategies showed similar performances in 3D lip movement tracking with a mean *ρ*_3*D*_ of 0.93 (no constraint), 0.93 (*act*_*all*_), and 0.92 (*act*_*rel*_), while the correlation with the normalised sEMG features varied: 0.27 (no constraint), 0.44 (*act*_*all*_), and 0.83 (*act*_*rel*_), respectively, illustrating different activation strategies. The forward solution was created with *act*_*rel*_, leading to good correlations in the experiment with *act*_*rel*_ constraint (mean $\rho =0.83$). Like in experiment I, the correlations were not perfect because of the other cost terms in the objective function and because of the noise added by integration.

Although we cannot perform statistical tests that will be reliable because of our small data set, some clear trends can be seen. Looking at the RMS errors, we note that the *e*_*pos*_ of no sEMG constraint was about the same as with *act*_*all*_ constraint, whereas for *act*_*rel*_ the *e*_*pos*_ was always higher than the other two. The activations errors *e*_*act*_ were always lower for *act*_*rel*_ constraint than the other two constraint, except for OOM and BUC. More surprisingly, the *act*_*rel*_ constraint resulted in a higher *e*_*pos*_, while we had expected the most accurate results from the use of *act*_*rel*_ as it was used in the forward simulation. Presumably, the influences of other cost terms and integration and the optimisation of muscle stress must have caused inaccuracies that resulted in better (though not perfect) estimated activations, sacrificing a little in motion tracking performance.

#### Conclusion

The ideal $em{g}_{val}=5\times {10}^{-4}$ enabled a reasonable sEMG-assisted IM appraoch. The sEMG cost term improved the correlations of activations as well as RMS errors while sacrificing only little in motion tracking performance.

### Experiment III: Inverse modelling using measured data

#### Goal and experimental set-up

The goal of experiment III was to apply our new sEMG-assisted IM approach on real data and test its performance. To do so, we used measurement data obtained from healthy volunteers. The motion targets were obtained from recorded position data registered to the generic face model with equation (10). The sEMG term’s penalty matrix **E** was set to 0 in case of no sEMG constraint and to $em{g}_{val}=5\times {10}^{-4}$ in case of the sEMG constraint *act*_*all*_ (as determined during the previous experiment, see Fig. 4).

#### Results

**Figure 6:**
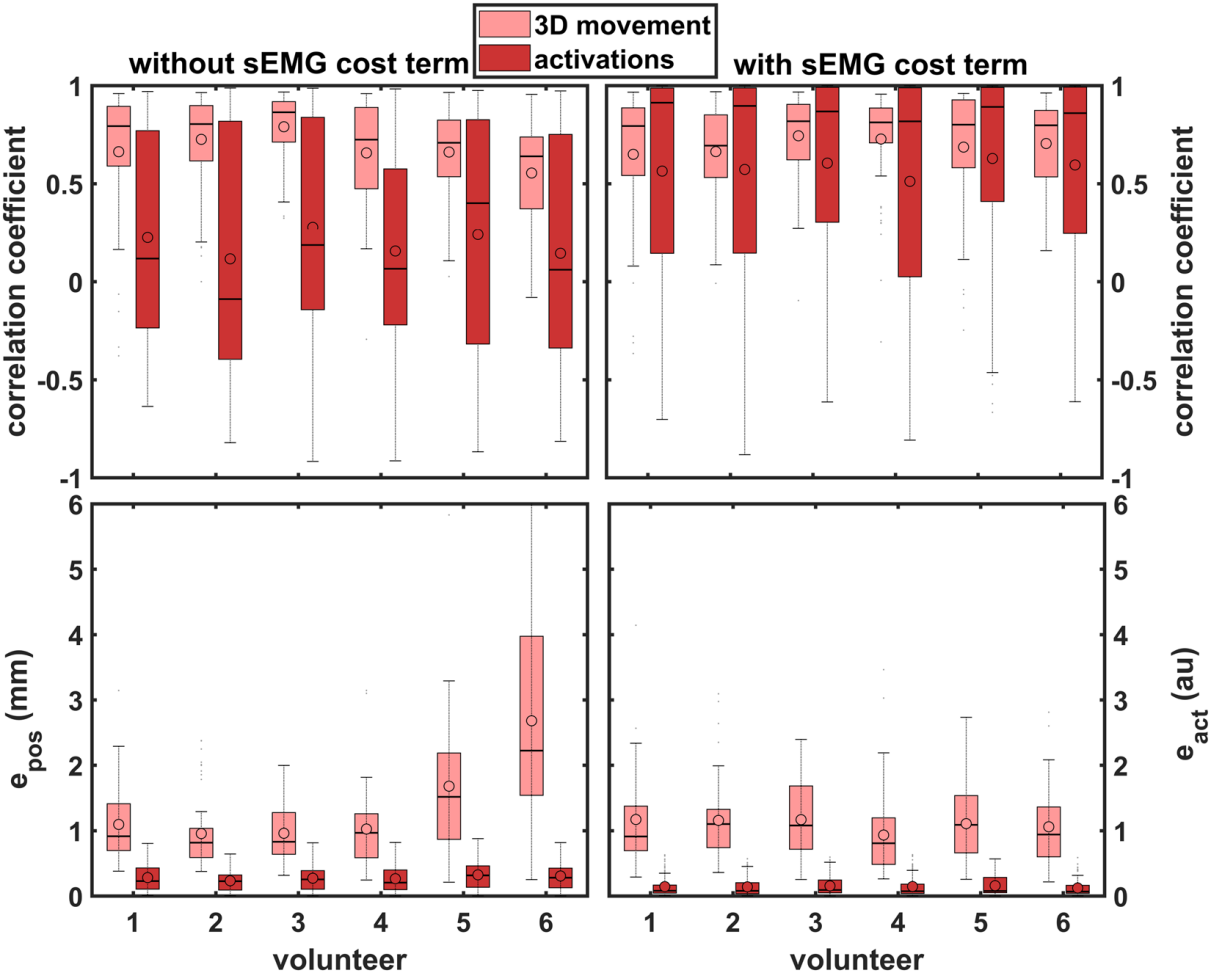
RMS errors and 3D correlation coefficients (movement) and Pearson’s correlation coefficients (activations) of the different volunteers for experiment III with and without sEMG cost term. The median is shown with a horizontal line and the mean with a dot. The boxes give first to third quartiles and the outer horizontal lines reflect minimum and maximum values (‘au’ is for ‘arbitrary units’).

Tables 1 and 2 show the RMS errors and the correlation coefficients, respectively. Congruence between measured muscle activations and calculated activations via inverse modelling was similar between volunteers, showing huge standard deviations and a mean around zero in correlations when using no sEMG constraint and reasonable to high correlations using *act*_*all*_ (Fig. 6). 3D movement correlations were similar, too. Remarkably, when using no constraint we found that volunteer 6 showed a deviating higher error in the movement *e*_*pos*_ (Fig. 6). The *ρ*_3*D*_ s of lip movement were always equal or higher compared to no constraint. Except for the marker 7. The mean *ρ*_3*D*_ s showed a moderate to good correlation ($\rho  \sim 0.7$). The *e*_*pos*_ was always lower in the sEMG-assisted approach, suggesting that the IM without constraint got stuck in a local minimum.

Calculating correlation coefficients for lip marker performance, we found that the lateral lip markers 1, 2, 6, and 7 performed better than the centre markers, similarly to the forward modelling results^7^. This can be explained by the fact that the volunteers’ centre markers moved notably, whereas the model’s centre markers only slightly deviated from their original position due to symmetry in the model. However, when we compare the *e*_*pos*_ for all lip markers we observe the opposite effect: the RMS errors are higher for the lateral markers than for the centre markers. This may also be explained by the fact that more movement allows for greater error due to a larger possible distance.

There was a lack of correlation without the sEMG constraint for the activations, caused by too many degrees of freedom in the muscle space. The sEMG-assisted inverse-modelling approach showed clear tendency of producing better, realistic and consistent muscle activations patterns.

Zooming in on the errors and correlation coefficients of the activations, those muscles whose activations were derived from measured muscles (DLI, BUC, LAO) performed worse than the muscles that were measured directly. This helps to explain why our forward model showed lower correlation coefficients in previous studies^7^. The OOP and OOM, derived from OOS and OOI measurements, also showed lower correlations (values), $\rho  \sim 0.5$ versus $\rho  \sim 0.7$. This is actually an interesting result, suggesting that the measurements do contribute a lot and can provide useful information. It would be interesting to look into the effects of only tracking the measured muscles instead of using derived muscle activations as we did here and to compare the results with experiments in which the DLI, BUC, and LAO are also measured directly.

#### Conclusion

In conclusion, adding sEMG tracking does not reduce 3D movement tracking accuracy, whilst giving better solutions in muscle activation tracking, as we already expected after experiments I and II. In essence, adding sEMG tracking tailors the inverse solution to a personalised activation strategy with equal performance. Apparently, surface EMG is sufficiently accurate without requiring any invasive needle approaches. However, challenges remain, as the inversion without constraint gave some questionable results, suggesting that the inversion may have got stuck in a local minimum. This would mean that including the sEMG constraint would be a way to avoid the inversion getting stuck in that miminum. However, it also hampers the general goal of seeking compensatory mechanisms by means of other muscle activation strategies. Also, because of a small dataset no statistical test could be performed. However, clear trends were observed and should be confirmed by future experiments.

### General results

Muscle stress varied per volunteer, per instruction, and per experiment (Table 3). Variation was highest between instructions and between experiments. The required computational time varied across simulations. Experiment III without the sEMG constraint may serve as a good example for computational times, as it was run completely on one workstation whereas the other experiments were distributed over the two workstations and the laptop computer, requiring longer computational times per simulation.

**Table 3:** Maximum muscle stress and computational times.

	sEMG constraint	Maximum muscle stress µ (σ) [kPa]	Computational time µ (σ)
EXPERIMENT II	Without	$$\begin{array}{cc}6.5\times {10}^{4} & (5.1\times {10}^{4})\end{array}$$	$$\begin{array}{cc}{\rm{06h06m55s}} & (\mathrm{00h\; 57m\; 09s})\end{array}$$
	*act*_*all*_	$$\begin{array}{cc}3.2\times {10}^{4} & (2.6\times {10}^{4})\end{array}$$	$$\begin{array}{cc}{\rm{11h04m17s}} & (\mathrm{05h\; 57m\; 01s})\end{array}$$
	*act*_*rel*_	$$\begin{array}{cc}6.8\times {10}^{4} & (5.7\times {10}^{4})\end{array}$$	$$\begin{array}{cc}{\rm{07h41m32s}} & (\mathrm{4h\; 02m\; 17s})\end{array}$$
EXPERIMENT III	Without	$$\begin{array}{cc}3.3\times {10}^{4} & (2.5\times {10}^{4})\end{array}$$	$$\begin{array}{cc}{\rm{05h31m15s}} & (\mathrm{06h\; 45m\; 46s})\end{array}$$
	*act*_*all*_	$$\begin{array}{cc}3.3\times {10}^{4} & (2.7\times {10}^{4})\end{array}$$	$$\begin{array}{cc}{\rm{07h24m35s}} & (\mathrm{04h\; 46m\; 19s})\end{array}$$

### General discussion

To our knowledge, this is the first study to describe the feasibility of sEMG-assisted inverse modelling of 3D lip movements using a biomechanical model of the face and lips. We have shown that implementing a simple sEMG cost term can direct the calculated muscle activations towards the derived muscle activations calculated from sEMG measurements. Adding the sEMG cost term showed a clear trend towards superior overall performance with regard to 3D lip marker trajectories as well as muscle activation patterns when compared with regular inverse modelling.

Our inverse-modelling approach has inherited the limitations of the model described by Eskes *et al*.^7^. First and foremost, the generic model does not account for individual physical geometry. Although our volunteers’ measurements were entered into the model initially, inaccuracies could build up during simulations due to mismatches in patient and model morphology. To account for individual geometry and anatomy, our future models should use the mismatch-and-repair algorithm or similar methods^48,49^, including diffusion-tensor magnetic resonance imaging (DT-MRI) to reveal muscle fibres and their trajectories^50^. Such combined approach may yield better approximation of muscle dimensions, orientations, and trajectories.

Furthermore, we may improve our simple skin model by introducing anisotropicity and viscoelasticity. Although the simplified soft representation does induce inaccuracies, these are negligible in the light of the larger errors caused by suboptimal registration and sEMG to force mapping. Our conclusions would probably not change if we would use more advanced models with anisotropic and viscoelastic properties.

Inverse modelling without sEMG tracking resulted in estimated activation patterns that totally lacked any correlation with the sEMG signals measured. It may even got stuck in a local minimum. Future experiments to address this could use the sEMGs as starting point and from there calculate the inverse activations. As expected, adding sEMG tracking gave calculated muscle activation patterns that resembled the measurements more closely. Pitermann *et al*. already highlighted the load-sharing problem by demonstrating that their calculated muscle activations patterns did not show any correlation with the measured intra-muscular rectified and integrated EMG patterns^36^. Varying the initial conditions resulted in different solutions to the inverse problem, including solutions with negative muscle activity. To address this issue, they restricted the inverted EMG to positive values, only, but they found no significant difference in performance between the methods with and without this positive constraint. This illustrates the difficulty of getting volunteer-specific muscle activation patterns when muscle redundancy causes an ill-posed inverse-dynamics problem. Nevertheless, they produced good correlation coefficients for 3D lip marker coordinates^36^, even when they applied a volunteer-specific face model to a different volunteer and restricted registration to general linear scaling.

These promising results encouraged us to make the step towards patient-friendly measurements. Pitermann’s team measured intramuscular EMG using invasive needle electrodes, but we chose to acquire muscle activation signals with the noninvasive technique of sEMG. Another improvement we made in the experimental set-up was measuring sEMG and 3D lip markers bilaterally. Pitermann *et al*. measured EMG on the left and facial movement on the right side, which may have induced error as volunteers may not have performed each instruction with perfect symmetry. Our results suggest that surface EMG is sufficiently accurate to replace the invasive technique of intramuscular EMG with intramuscular needle placement.

Terzopoulos & Waters created one of the first physics-based face models using discrete mass-spring systems to estimate muscle activity from video employing interactive deformable contours (snakes)^33^. They were able to resynthesize facial expression from estimated muscle activity using a simple, yet powerful algorithm, which called for further research in this direction. Where they mapped static facial expression to muscle activity in 2D, our results relate to 3D musculature. Incorporating improved tissue biomechanics, the ArtiSynth model uses a continuum mechanics based FE formulation as well as an advanced orbicularis oris muscle, in contrast to the two fiducial points used in Terzopoulos & Waters’ model. Furthermore, we increased the number of perioral muscles to 20, where Terzopoulos & Waters studied merely 4.

Kim & Gomi and Kim *et al*. created a discrete model of lumped nodal masses connected via viscoelastic elements^34,35^. Despite much lower computational costs, a major drawback of their set-up is the simplified representation of reality provided by their continuum-based finite-element model. Moreover, their inverse-modelling approach involved a gradient descent search with optimisation per trial instead of per sample and without quantitative reporting. However, if sufficiently accurate, such model may be a useful addition to our virtual-therapy toolbox for rapidly simulating new inverse solutions. Our computational times, were quite high, especially when simulating the instruction set proposed in Eskes *et al*. for all essential functional movements^6^.

To exert similar force on the elements in the model across experimental conditions, maximum muscle stress had to be variable. Although muscle stress differed per volunteer and per instruction, we found that mean muscle stress was similar in experiments II and III, at $3.3\times {10}^{4}\,{\rm{kPa}}$. The variance can be explained by the fact that muscle activation amplitudes differed, as did the extent of co-contraction. The different amplitudes may be explained by sEMG-technical issues. Signal amplitude may have been affected by numerous factors including sensor placement^51^: inaccurate sensor placement will inevitably contribute to crosstalk.

Another important paper by Hirayama *et al*.^52^ reports on inverse dynamics of articulatory trajectories. Using a supervised-learning algorithm, they followed the direct inverse-modelling approach as described by Jordan & Rumelhart^53^. However, theirs was a statistical model, while we prefer biomechanical models that also account for physical laws to simulate the effects of surgical interventions.

All of the above publications confirm the difficulty of validating computed muscle activations with the actual muscle activation strategy. Most researchers have used EMG data as reference values to test algorithm performance. This method is even less reliable when EMG information is used to best track the muscle activation patterns. Recently, Nikooyan *et al*. reported on a new method to validate forces (and activation levels) in patients with shoulder prostheses, measuring the glenohumeral-joint reaction forces *in vivo* ^29^. Similar data obtained with knee prostheses were made available for the “Grand Challenge Competition to Predict *In Vivo* Knee Loads”^54,55^. Unfortunately, this type of direct-force data cannot be obtained for facial muscles.

Despite these challenges, we were able to demonstrate that performance in 3D movement tracking did not decrease drastically - in fact, it had a tendency towards improvement - while the activation tracking improved. We think this will open new ways of obtaining realistic person-specific activation strategies.

## Conclusion

We have demonstrated the feasibility of an sEMG-assisted inverse-modelling algorithm for the perioral region. Our method means an important step in the development of a virtual-surgery toolkit for the preoperative estimation of function loss after lip and oral cavity cancer surgery.

### Ethical approval

All volunteers were informed about the experiment and about their rights. Written consent was obtained for publishing the photograph in Fig. 1. The Medical Research Ethics Committee (MREC) of the Netherlands Cancer Institute determined that the study did not fall under the scope of the Medical Research Involving Human Subjects Act (WMO), because the study did not infringe the (psychological) integrity of the volunteers. The measurements were noninvasive and not stressful. Thus, prior review by an accredited MREC was not required. The study was performed within the Dutch legislation regarding the Agreement on Medical Treatment Act, Personal Data Protection Act, and the Code of Conduct for Responsible Use of the Federa (Dutch Federation of Biomedical Scientific Societies). Written informed consent was obtained.
